# Sinoatrial node pacemaker cells share dominant biological properties with glutamatergic neurons

**DOI:** 10.1007/s13238-020-00820-9

**Published:** 2021-02-06

**Authors:** Dandan Liang, Zhigang Xue, Jinfeng Xue, Duanyang Xie, Ke Xiong, Huixing Zhou, Fulei Zhang, Xuling Su, Guanghua Wang, Qicheng Zou, Yi Liu, Jian Yang, Honghui Ma, Luying Peng, Chunyu Zeng, Gang Li, Li Wang, Yi-Han Chen

**Affiliations:** 1 Department of Cardiology, East Hospital, Tongji University School of Medicine, Shanghai, 200120 China; 2 Key Laboratory of Arrhythmias of the Ministry of Education of China, Tongji University School of Medicine, Shanghai, 200120 China; 3 Institute of Medical Genetics, Tongji University, Shanghai, 200092 China; 4 Department of Regenerative Medicine, Tongji University School of Medicine, Shanghai, 200092 China; 5 Reproductive Medicine Center, Tongji Hospital, Tongji University School of Medicine, Shanghai, 200065 China; 6 Department of Pathology and Pathophysiology, Tongji University School of Medicine, Shanghai, 200092 China; 7 Department of Cardiology, Daping Hospital, Chongqing, 400042 China; 8 Department of Neurology, East Hospital, Tongji University School of Medicine, Shanghai, 200120 China; 9 State Key Laboratory of Cardiovascular Disease, Fuwai Hospital, National Center for Cardiovascular Diseases, Chinese Academy of Medical Sciences and Peking Union Medical College, Beijing, 100037 China

**Keywords:** sinoatrial node, pacemaker cell, glutamatergic neuron, single-cell RNA-seq, electrophysiology

## Abstract

Activation of the heart normally begins in the sinoatrial node (SAN). Electrical impulses spontaneously released by SAN pacemaker cells (SANPCs) trigger the contraction of the heart. However, the cellular nature of SANPCs remains controversial. Here, we report that SANPCs exhibit glutamatergic neuron-like properties. By comparing the single-cell transcriptome of SANPCs with that of cells from primary visual cortex in mouse, we found that SANPCs co-clustered with cortical neurons. Tissue and cellular imaging confirmed that SANPCs contained key elements of glutamatergic neurotransmitter system, expressing genes encoding glutamate synthesis pathway (*Gls*), ionotropic and metabotropic glutamate receptors (*Grina*, *Gria3*, *Grm1* and *Grm5*), and glutamate transporters (*Slc17a7*). SANPCs highly expressed cell markers of glutamatergic neurons (*Snap25* and *Slc17a7*), whereas *Gad1*, a marker of GABAergic neurons, was negative. Functional studies revealed that inhibition of glutamate receptors or transporters reduced spontaneous pacing frequency of isolated SAN tissues and spontaneous Ca^2+^ transients frequency in single SANPC. Collectively, our work suggests that SANPCs share dominant biological properties with glutamatergic neurons, and the glutamatergic neurotransmitter system may act as an intrinsic regulation module of heart rhythm, which provides a potential intervention target for pacemaker cell-associated arrhythmias.

**Supplementary information:**

The online version of this article (10.1007/s13238-020-00820-9) contains supplementary material, which is available to authorized users.

## INTRODUCTION

Heartbeats are triggered by electrical impulses generated by the SAN (Mangoni and Nargeot, 2008; Lakatta et al., 2010; Cingolani et al., 2018). SAN dysfunction can lead to bradycardia, cardiac arrest, syncope or even sudden cardiac death (Ewy, 2014; Clauss et al., 2019). Due to lack of comprehensive understanding of the complex tissue architecture and cellular diversity of the SAN, few treatment options are available for arrhythmias caused by SAN dysfunction, including sinus bradycardia, sinus arrest, sinus atrial block and sick sinus syndrome (Chandler et al., 2009; Ewy, 2014; Ritter et al., 2015). Pacemaker cells, the parenchymal cells in the SAN, are one of the most poorly defined entities in the heart. It is now believed that the coupled-clock system, composed of surface membrane voltage clocks and intracellular Ca^2+^ clocks is responsible for igniting periodically propagable action potentials in SANPCs (Chandler et al., 2009; Lakatta et al., 2010; Linscheid et al., 2019). Interestingly, cortical neurons also possess the intrinsic ability to generate spontaneous electrical pulses (Pulver and Griffith, 2009; O’Leary et al., 2014; Morquette et al., 2015). This shared functional property hints at mechanistic similarities between SANPCs and cortical neurons.

In the present study, we compared single-cell transcriptome of mouse SANPCs with that of primary visual cortex cells (CCs), and found that SANPCs co-clustered with neuronal cells. SANPCs expressed not only cell markers of glutamatergic neurons, but also key components of the glutamatergic neurotransmitter system. Functional studies revealed that antagonists of glutamate receptors or transporters dramatically decreased the spontaneous pacing frequency of isolated SAN tissues and the frequency of spontaneous Ca^2+^ transients in single SANPC. Collectively, our study demonstrates that SANPCs and glutamatergic neurons share both cellular and functional properties.

## RESULTS

### Co-clustering of SANPCs with cortical neurons at the single-cell transcriptomic resolution

**Figure 1:**
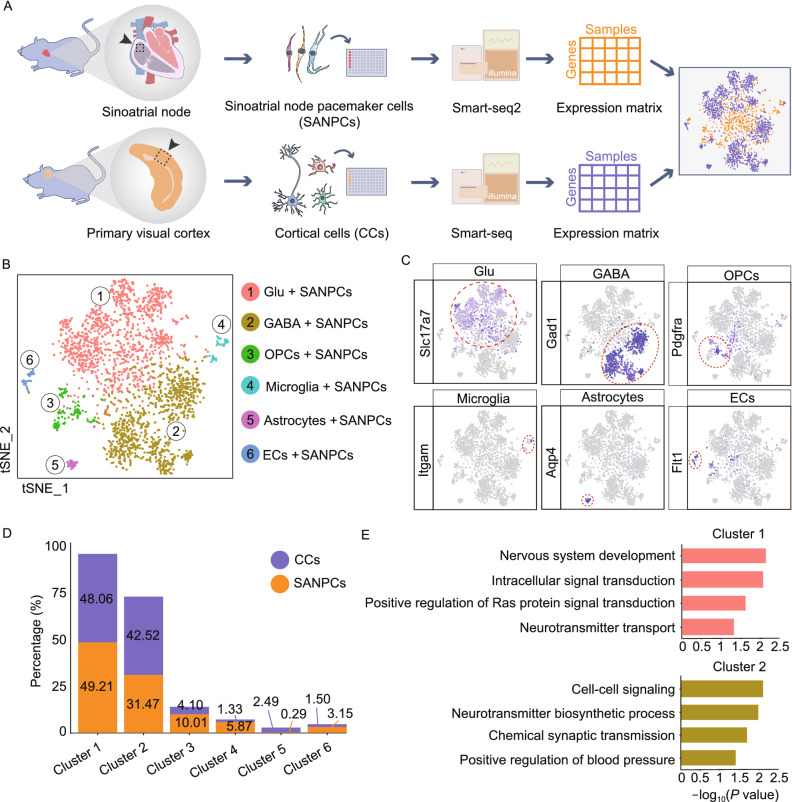
**SANPCs co-clustered with cortical neurons at single-cell transcriptomic resolution**. (A) Schematic illustration of the strategy for single-cell datasets comparison of mouse SANPCs and primary visual CCs. (B) t-SNE visualization of integrated single-cell transcriptomic analysis of SANPCs (*n* = 699) and CCs (*n* = 1,806). Six cell clusters were identified. (C) Clusters were identified according to the expression of canonical cell markers in CCs. *Slc17a7*, glutamatergic neuron (Glu); *Gad1*, GABAergic neuron (GABA); *Pdgfra*, oligodendrocyte precursor cells (OPCs); *Itgam*, microglia; *Aqp4*, astrocytes; *Flt1*, endothelial cells (ECs). (D) Distribution percentages of SANPCs and CCs in each cell cluster. (E) GO analysis showing enriched biological processes in Cluster 1 and Cluster 2

To provide an overall view of SANPCs and CCs, we profiled the single-cell transcriptome analysis of 718 SANPCs from 21 adult mice. With Seurat, we compared these SANPCs with 1,809 CCs, which contained diverse cell types, including glutamatergic neurons, GABAergic neurons and non-neuronal cells (Tasic et al., 2016) (Fig. 1A). These two single-cell transcriptomic datasets were filtered and the batch effects were removed by Seurat canonical correlation analysis (Butler et al., 2018). A total of 2,505 SANPCs and CCs passed quality control and were assigned into six distinct cell clusters (Cluster 1–6) (Fig. 1B), in which the SANPCs were confirmed by the expression of SANPC marker gene *Hcn4* (Fig. S1A and S1B). Of these, Clusters 1 and 2 each contained three transcriptomic cell subpopulations (Fig. S1C). Based on the expression of canonical markers in the nervous system, we observed that the entire cell population could be segregated into distinct cell types, corresponding to glutamatergic neurons (*Slc17a7*^+^), GABAergic neurons (*Gad1*^+^), oligodendrocyte precursor cells (*Pdgfra*^+^), microglia (*Itgam*^+^), astrocytes (*Aqp4*^+^) and endothelial cells (*Flt1*^+^) (Fig. 1B and 1C). Notably, SANPCs co-clustered with CCs in each cluster, especially in clusters identified as neurons (Figs. 1B,1C and S1B). There were 80.68% SANPCs that co-clustered with glutamatergic neurons (Cluster 1, 49.21%) and GABAergic neurons (Cluster 2, 31.47%), while the rest co-clustered with non-neuronal cells (Fig. 1D), suggesting a potential relationship between SANPCs and CCs, particularly with glutamatergic neurons and GABAergic neurons. The cell subpopulations in Cluster 1 and Cluster 2 expressed specific cell subtype markers of glutamatergic neurons and GABAergic neurons (Fig. S1D). The greatest subpopulation of SANPCs was identified in Cluster 1 (34.48%), which were *Enpp2*^+^ glutamatergic neurons (Fig. S1E).

Gene Ontology (GO) enrichment analysis of differentially expressed genes (DEGs) indicated that the DEGs enriched in each cell cluster corresponded to their characteristic biological processes. For example, Cluster 1 was associated with intracellular signal transduction and neurotransmitter transport. Cluster 2 expressed genes involved in cell-cell signaling and chemical synaptic transmission (Fig. 1E), while enriched genes for other clusters were related to non-neuronal and cardiac functions (Fig. S2).

### The identification of glutamatergic neurotransmitter system in SANPCs

**Figure 2:**
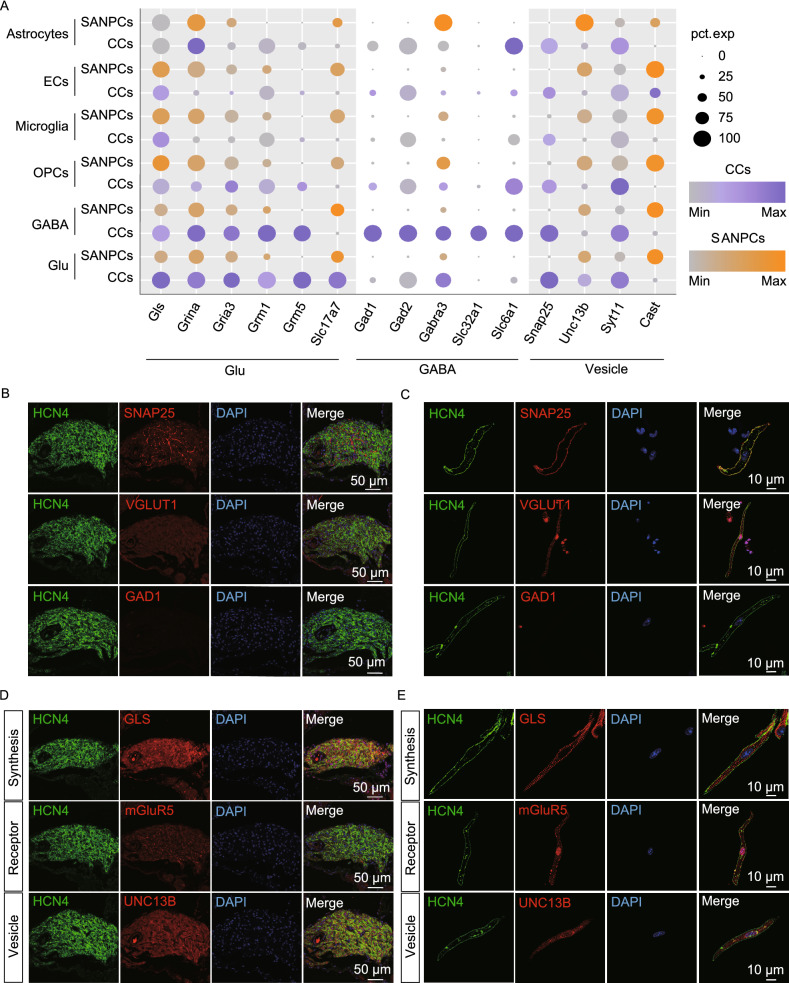
**The SANPCs expressed molecular basis of glutamatergic neurotransmitter system**. (A) Expression of functional genes related to glutamate synthesis, reception and transport in SANPCs and CCs in each cluster. The size of the dots showed the percentage of cells which expressed the genes in clusters. The brightness of colors showed the relative expression level of genes. (B and C) Immunofluorescence staining of the expression of glutamatergic and GABAergic neuron markers at the tissue (B) and single cell (C) level. (D and E) Immunofluorescence staining of the expression of the glutamate synthesis marker GLS (*Gls*) and glutamate receptor mGluR5 (*Grm5*), as well as synaptic vesicle gene UNC13B (*Unc13b*) in SAN tissue (D) and single SANPC (E). Glu, glutamatergic neuron; GABA, GABAergic neuron; OPCs, oligodendrocyte precursor cells; ECs, endothelial cells

Co-clustering of SANPCs with neurons suggested molecular similarity between the two. Synthesis, transport and reception of neurotransmitters underlie the major functions of neurons (El Mestikawy et al., 2011). To determine the specific characteristic of neuronal property of SANPCs, we analyzed the expression of key genes associated with neurotransmitter systems in SANPCs (Fig. 2A). Our results demonstrated that SANPCs contained key elements of glutamatergic neurotransmitter system, expressing genes encoding glutamate synthesis pathway (*Gls*), ionotropic and metabotropic glutamate receptors (*Grina*, *Gria3*, *Grm1* and *Grm5*), and glutamate transporters (*Slc17a7*) (Fig. 2A). The expression of these glutamatergic neuronal elements was further confirmed in mouse SAN sections and isolated single SANPC by immunofluorescence (Figs. 2B–E and S3). By contrast, the genes critical to the function of GABAergic neurons (e.g., *Gad1*, *Gad2*, *Gabra3*, *Slc32a1*, *Slc6a1*) were lowly expressed in SANPCs (Fig. 2A).

We also detected the protein expression of cell marker genes for both glutamatergic neurons and GABAergic neurons, including presynaptic plasma membrane protein SNAP25 (*Snap25*), vesicle-bound glutamate transporter VGLUT1 (*Slc17a7*), and glutamate decarboxylase GAD1 (*Gad1*) in SAN sections and single SANPC by immunofluorescence. Our results confirmed the expression of SNAP25 and VGLUT1 in SANPCs, while the expression of GAD1 was not observed (Fig. 2B and 2C), suggesting that SANPCs displayed properties of glutamatergic neurons (*Snap25*^+^, *Slc17a7*^+^ and *Gad1*^−^) rather than GABAergic neurons (*Snap25*^+^, *Slc17a7*^−^ and *Gad1*^+^).

In addition, the genes associated with synaptic vesicle cycle (*Unc13b*, *Syt11* and *Cast*) were also highly expressed in SANPCs (Figs. 2A, 2D, 2E and S3). Taken together, these results demonstrate that SANPCs possess molecular and cellular properties of glutamatergic neurons.

### Inhibition of glutamate receptors or transporters reduced spontaneous pacing frequency of isolated SAN

Glutamate is the major excitatory neurotransmitter in the central nervous system, whose signaling depends on specialized receptors including ionotropic glutamate receptors (iGluRs), acting as glutamate-gated ion channels, and metabotropic glutamate receptors (mGluRs), acting via G proteins. iGluRs are classified into three major subtypes: N-methyl-d-aspartate receptors (NMDARs), α-amino-3-hydroxy-5-methyl-4-isoxazole propionic acid receptors (AMPARs) and kainate receptors (Nedergaard et al., 2002; Divito and Underhill, 2014; Malik and Willnow, 2019).

**Figure 3:**
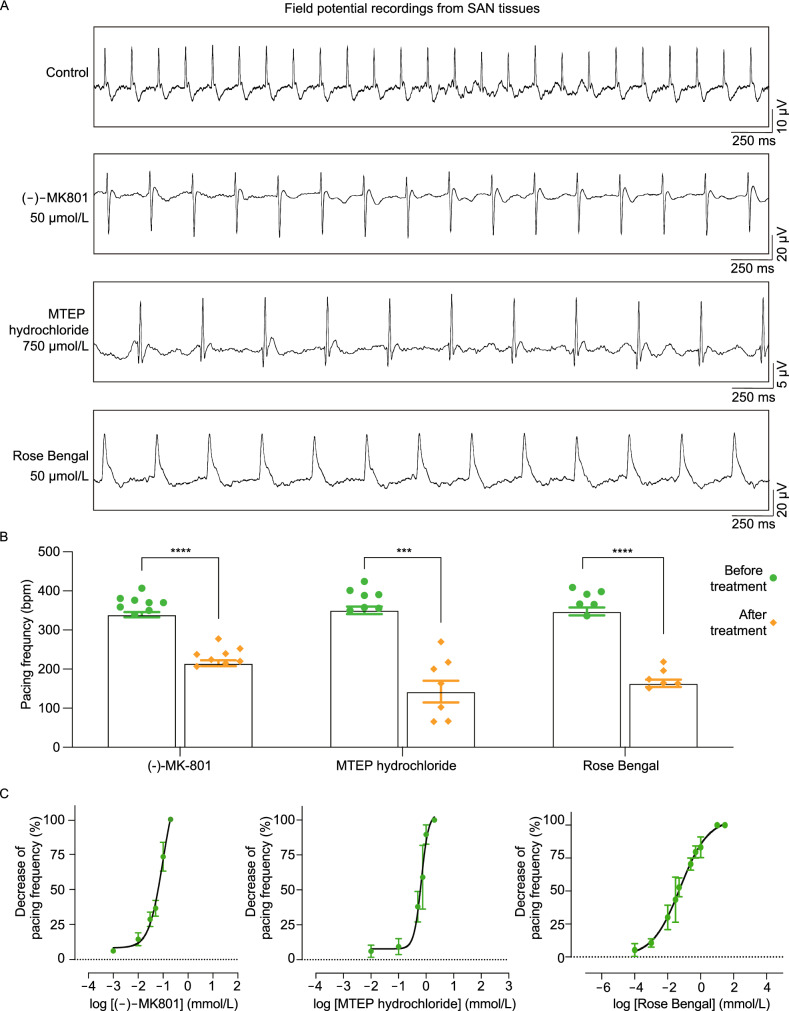
**Glutamate receptor or transporter antagonists reduced spontaneous pacing frequency of SAN tissues in a concentration-dependent manner**. (A) Representative tracings of field potential in the vehicle control and antagonist treatment groups. (B) Quantification of spontaneously pacing frequency alteration in SAN tissues under (-)-MK801 (50 μmol/L), MTEP hydrochloride (750 μmol/L) or Rose Bengal (50 μmol/L) treatment (*n* = 6–8/group, the *P* value was calculated by unpaired *t* test, ***P < 0.001, ****P < 0.0001). bpm, beats per minute. (C) Concentration-response curves of antagonist-induced alterations in spontaneous pacing frequency of SAN tissues

To explore functional glutamatergic properties of SANPCs, we evaluated the effect of glutamate receptor antagonists on pacing frequency of SAN tissues dissected from the heart. We found that (-)-MK801, a non-competitive NMDAR antagonist, and MTEP hydrochloride, a non-competitive mGluR5 receptor (a subtype of mGluRs) antagonist, caused a concentration-dependent decrease in spontaneous pacing frequency of SAN tissues (Fig. 3A–C). The median effective inhibitory concentrations (IC_50_) were 97.23 μmol/L (95% confidence interval (CI): 65.51–157.6), (-)-MK801) and 668.3 μmol/L (95% CI: 587–760.9, MTEP hydrochloride) (Fig. 3C). The pacing frequency of SAN tissues in control group was 342.14 ± 23.13 beats per minute (bpm). The value after antagonist treatment was significantly reduced to 215 ± 21.12 bpm ((-)-MK801) and 142.43 ± 73.05 bpm (MTEP hydrochloride), respectively (Fig. 3B).

The vesicular glutamate transporters (VGLUTs) are important in the removal and uptake of glutamate from the extracellular space, playing a critical role in the regulation of glutamatergic neurotransmission in the central nervous system (Schenck et al., 2009). We therefore investigated the effect of the VGLUT inhibitor Rose Bengal on the regulation of SAN rhythm. Application of Rose Bengal resulted in a concentration-dependent reduction of pacing frequency as shown by field potential recordings from SAN tissues (IC_50_ = 58.52 μmol/L, 95% CI: 37.51 to 91.05) (Fig. 3). The pacing frequency of SAN tissues was reduced to 163.5 ± 22.93 bpm after Rose Bengal treatment (Fig. 3B). Taken together, our data revealed that the glutamate receptors and transporters participate in the regulation of autonomic rhythm in SAN.

### Inhibition of glutamate receptors or transporters diminished pacemaking activity of single SANPC

**Figure 4:**
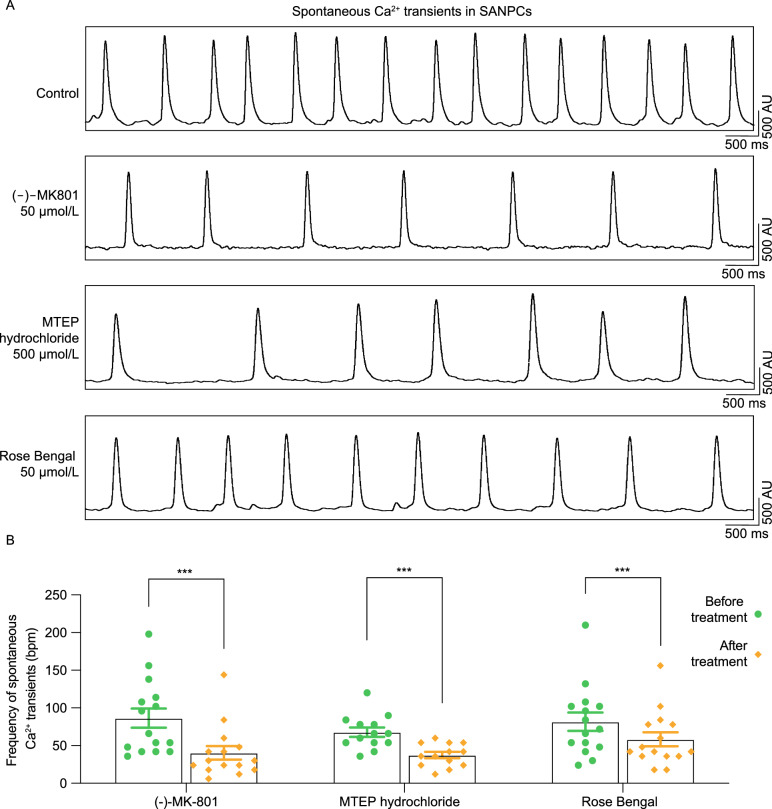
**Glutamate receptor or transporter antagonists decreased the frequency of spontaneous Ca**^**2+**^ **transients in SANPCs**. (A) Representative traces of Ca^2+^ transients in SANPCs during the application of glutamate receptor or transporter antagonists. (B) Statistics of the frequency of spontaneous Ca^2+^ transients in SANPCs under (-)-MK801 (50 μmol/L), MTEP hydrochloride (500 μmol/L) or Rose Bengal (50 μmol/L) treatment (*n* = 11–15 cells from six mice per group). The *P* value was calculated by the mixed-effects model with Bonferroni’s multiple comparison post-test (***P < 0.001)

It is well known that the intervention targeting glutamatergic neurotransmitter system can alter intracellular calcium dynamics of glutamatergic neurons, which in turn brings nerve excitation. Intracellular Ca^2+^ release from endoplasmic reticulum decides the pacemaker activity of SANPCs (Mangoni et al., 2003; Vinogradova et al., 2008, 2010). Thus, by using the time-lapse high-speed imaging of intracellular calcium in fluo-4 loaded SANPCs, we evaluated the effects of glutamatergic tool drugs on SANPCs automaticity. Consistent with the findings in SAN tissues, a non-competitive NMDAR antagonist (-)-MK801 and a non-competitive mGlu5 receptor antagonist MTEP hydrochloride decreased the frequency of spontaneous Ca^2+^ transients in single SANPC (Fig. 4). In addition, the frequency of spontaneous Ca^2+^ transients also significantly slowed down in the presence of Rose Bengal (Fig. 4). The frequency of spontaneous Ca^2+^ transients of SANPCs in control group was 78 ± 33.83 bpm, while the value in antagonist treatment groups was reduced to 48.96 ± 22.52 bpm (MK801), 37.38 ± 15.33 bpm (MTEP hydrochloride) and 58.4 ± 36.39 bpm (Rose Bengal), respectively (Fig. 4B).

Since it’s been documented that frequency of spontaneous Ca^2+^ transients to some extent reflects the pacing activity of cardiac pacemaker cells, these results suggest that the glutamatergic neurotransmitter system can regulate the spontaneous rhythmic activity of the SANPCs. Collectively, our results revealed that the SANPCs have functional properties of glutamatergic neurons at single-cell level.

## DISCUSSION

Our current study reveals that the SANPCs possess the biological properties of glutamatergic neurons. First, SANPCs co-clustered with neurons of the primary visual cortex at a single-cell transcriptomic resolution. Second, SANPCs expressed the cell markers of glutamatergic neurons. Third, SANPCs contained key elements of neurotransmitter system of glutamatergic neurons. Finally, the automaticity properties of SANPCs were sensitive to the classical tool drugs acting on the glutamatergic neurons.

Although SANPCs have not been reported to possess a functional glutamatergic neurotransmitter system, it is already known that SANPCs and neurons have broad commonalities in electrophysiology (O’Leary et al., 2014; Murphy and Lazzara, 2016). Both SANPCs and neurons have excitability and conductivity (Kalmbach et al., 2018). They generate spontaneously propagative action potentials (excitations), which are transmitted between cells (conductions). Ion channels that form the action potentials of the two types of cells are similar. HCNs families are expressed in both SANPCs and neurons (Stieber et al., 2003; Baruscotti et al., 2011; Kalmbach et al., 2018). However, although there are similarities between the two types of cells, no systematic in-depth comparison has ever been made, and we speculate that the main obstacle lies in the small number of SANPCs. Through scRNA-seq and electrophysiological techniques, we revealed the glutamatergic neuron properties of SANPCs.

The discovery of the glutamatergic neuron property of SANPCs has potentially important implications for the treatment of SAN dysfunction. Given the presence of molecules such as glutaminase, receptors and transporters in SANPCs, it is reasonable to speculate that the glutamatergic neurotransmitter system may be a new target for the intervention of SAN diseases. Indeed, our results showed that inhibition of mGlu5 receptor, NMDAR or VGLUT1 transporter reduced the spontaneous pacing frequency of isolated SAN and decreased the pacing activity of single SANPC. Furthermore, because SANPC-like cells are also present in atrioventricular nodes and Purkinje fibres (Dun and Boyden, 2008; Boyden et al., 2016), it is possible that the glutamatergic neurotransmitter system may also be a potential viable target for the treatment of atrioventricular node or Purkinje fibre dysfunction.

For the single-cell RAN sequencing data analysis, the batch effect is a common issue when integrating different datasets. Systematic variations mainly result from the difference of technology platforms for library construction and sequencing, as well as single cell isolation methods and other variations (Haghverdi et al., 2018; Tran et al., 2020). In the present study, the batch effects between SANPCs and CCs datasets were unavoidable and obvious since the cells were isolated from different tissues and the single-cell transcriptomic data were analyzed based on different technology platforms (Fig. S4). Although Seurat V2 has good performance on the batch correction across different conditions and technologies (Butler et al., 2018; Tran et al., 2020), we also performed cross-validation about the batch correction using Seurat V3. The results showed that part of SANPCs co-clustered with glutamatergic neurons, confirming the close relationship between SANPCs and glutamatergic neurons (Fig. S5).

The development of SAN has been extensively investigated. Gene expression and genetic studies in mice have revealed transcriptional networks underlying the control of the heart rate and rhythm (van Eif et al., 2018). To explore whether embryonic SANPCs also share some properties with glutamatergic neurons, we compared the single cell transcriptome datasets of SANPCs from embryonic day 13.5 (E13.5) mice with the CCs used in our study (Li et al., 2019). Interestingly, similar to adult SANPCs, we found that part of embryonic SANPCs co-clustered with glutamatergic neurons (Fig. S6), suggesting the property of glutamatergic neurons have already existed in embryonic SANPCs. Notably, neither the embryonic SANPCs nor adult SANPCs co-clustered with GABAergic neurons using the Seurat V3 integrating single-cell transcriptomic data analysis software (Figs. S5 and S6), further demonstrating that SANPCs are more similar to glutamatergic neurons.

The cardiac autonomic nervous system, which is mainly subdivided into sympathetic and parasympathetic components, plays an important role in the modulation of SAN electrophysiology (Shen and Zipes, 2014; Finlay et al., 2017; Herring et al., 2019). Sympathetic stimulation increases sinus heart rate, potentially leading to significant sinus tachycardia, whereas parasympathetic stimulation reduces sinus heart rate, even causes cardiac arrest. In the present study, although high expression of adrenergic receptor and acetylcholine receptor were observed in SANPCs, other components of adrenergic and acetylcholine neurotransmitter system are barely detected, suggesting these two neurotransmitter systems are not complete in SANPCs (Fig. S7). We identified that the SANPC possessed its own glutamatergic neurotransmitter system, with its inhibition via antagonists producing pronounced effects on the spontaneous pacing frequency of SAN and the Ca^2+^ transient frequency of single SANPC, indicating that the glutamatergic neurotransmitter system may serve as an alternate target in the functional regulation of the SAN.

Autonomic activity is a hallmark of cardiac pacemaker cells that distinguishes them from non-pacemaker cells. It is accepted that the coupled-clock system, is in control of the autonomic rhythms of SANPCs (Mangoni and Nargeot, 2008; Lakatta et al., 2010; Cingolani et al., 2018). This coupled-clock is initiated by local calcium release from sarcoplasmic reticulum, which is largely controlled by intracellular Ca^2+^ concentrations, protein phosphorylation and energy metabolism (Vinogradova et al., 2008, 2010; Zhu et al., 2018). Multiple molecules synergize in the generation of auto-rhythmicity and periodic action potentials in SANPCs. Our findings demonstrated that the glutamatergic neurotransmitter system can regulate the autonomic activity. However, the specific pathway by which it exerts such effect remains to be further investigated.

The definition of cell properties is key to understanding its biological nature and physiological functions of SANPCs. Here, we identified a previously unknown cellular property of SANPCs, broadening the traditional concept that SANPCs are differentially specialized cardiac myocytes. Combining SANPCs and visual CCs transcriptome datasets, we defined six transcriptomic cell clusters at single-cell resolution. These cell clusters cover all major cell types in mouse primary visual cortices. Intriguingly, even within the population of SANPCs, different cell clusters expressed a distinct set of genes related to neurons and non-neurons, indicating that these clusters may play diverse roles in maintaining heart rhythm. It is therefore vital to systematically determine the function of SANPCs in distinct cell clusters in future studies.

In summary, our data suggest that SANPCs possess the cell properties of glutamatergic neurons. The glutamatergic neurotransmitter system identified in SANPCs may serve as the intrinsic regulation system of spontaneous bioelectrical activity of SAN, and may be potential intervention target of SAN diseases. These findings shed new light on the fundamental biology of SAN and the control of heart rhythm.

## MATERIALS AND METHODS

### Study design

We performed a comparative analysis of the gene expression between SANPCs and primary visual CCs at single-cell transcriptomic level. 718 mouse SANPCs were manually isolated for scRNA-seq using Smart-seq2 (PRJNA531288), while the data of 1,809 mouse cortical cells were obtained from other research group (GSE71585). Seurat clustering analysis was used to determine the transcriptomic relationship between individual cells, while the neuronal features of SANPCs and CCs were defined by the gene expression pattern of synthesis, receptors and transporters of neurotransmitters.

### Animals

This study conformed to the rules of the Guide for the Care and Use of Laboratory Animals made by the U.S. National Institutes of Health. All the animal experiments were approved by the Animal Care and Use Committee of Tongji University School of Medicine.

### SANPCs isolation

SANPCs were isolated from male C57BL/6 mice (8–12 weeks of age) as previously described (Lolicato et al., 2014). Briefly, the heart was removed quickly and placed in prewarmed Tyrode’s solution containing: 140 mmol/L NaCl, 5.4 mmol/L KCl, 1.2 mmol/L KH_2_PO_4_, 1.8 mmol/L CaCl_2_, 1.0 mmol/L MgCl_2_, 5.5 mmol/L glucose and 5 mmol/L HEPES (pH was adjusted to 7.4 with NaOH). The solution was continuously oxygenated with O_2._ The SAN was dissected from the region bordered by the crista terminalis and the superior and inferior vena cava. The SAN tissue was cut into small pieces and washed twice in low-Ca^2+^ solution containing: 140 mmol/L NaCl, 5.4 mmol/L KCl, 1.2 mmol/L KH_2_PO_4_, 0.2 mmol/L CaCl_2_, 18.5 mmol/L glucose, 50 mmol/L taurine and 1.0% BSA (pH was adjusted to 6.9 with NaOH). The pieces of SAN tissue were then digested at 36–37 °C for 20–25 min in low-Ca^2+^ solution containing elastase (0.3 mg/mL; Worthington, NJ, USA), collagenase type II (0.8 mg/mL; Worthington, NJ, USA) and protease (0.13 mg/mL; Sigma, Chemical Co.) with gentle agitation. The digestion was quenched by transferring the pieces to modified Kraftbruhe solution, containing: 100 mmol/L potassium glutamate, 10 mmol/L potassium aspartate, 25 mmol/L KCl, 10 mmol/L KH_2_PO_4_, 2 mmol/L MgSO_4_, 20 mmol/L taurine, 5 mmol/L creatine, 0.5 mmol/L EGTA, 20 mmol/L glucose, 5 mmol/L HEPES and 1.0% BSA (pH was adjusted to 7.2 with KOH).

### scRNA-seq and data processing

The scRNA-seq was performed using Smart-seq2 with minor modification. In brief, single SANPC was lysed in microtube, the released RNA was reversed to first-strand cDNA using Superscript III (18080044, Invitrogen, USA) and then amplificated using KAPA polymerase (KK2601, Kapa Biosystems, USA), The product was purified using Ampure XP Beads (A63881, Beckman Coulter, USA) for Nextera Tagmentation (FC-131-1096, Illumina, USA), then the libraries were sequenced with paired end 2 × 150 bp reads on Illumina HiSeq X10 to generated the raw sequencing data.

Raw data of scRNA-seq of SANPCs were initially trimmed with Trimmomatic (V0.33). Then the obtained clean reads were aligned to mouse reference genome (GRCm38), using Hisat2 (V2.0.5), and annotated using GENCODE (release 20). Finally, the gene expression was quantified by read counts.

### Integrated co-clustering analysis

Read count of SANPCs and CCs were used as input to Seurat V2 R package (V2.3.4) for the clustering analysis. The Seurat objects of SANPCs data and CCs data were generated using “CreateSeuratObject” function. Then “FilterCells” function was used for dropping the low-quality cells out which expressed less than 1000 or more than 12,500 genes. After the data were normalized and scaled by “NormalizeData” and “ScaleData” function, 2,000 high variable genes were calculated separately in SANPCs and CCs datasets and then merged into 3,290 unique high variable genes across two datasets. Subsequently, the batch effects of these two objects were removed and then combined into an integrated object using “RunCCA” and “AlignSubspace” function, basing on the canonical correlation analysis alignment in Seurat. 15 CC components were chosen for the dimensionality reduction according to the result of “MetageneBicorPlot” function, which examined a measure of correlation strength for each CC (Fig. S8). Then we performed tSNE analysis through “RunTSNE” and “FindClusters” function with 0.6 resolution. Diverse cell subpopulations were identified and annotated using the canonical neuronal cell markers.

We also used Seurat V3 R package (V3.2.2) for the batch effect correction between SANPCs data and CCs data with similar parameters. 2,000 variable genes were identified using “FindVariableFeatures” function for the PCA analysis. “FindIntegrationAnchors” function was used to integrate the two objects. Based on the results of “ElbowPlot” and “JackStraw” functions, 15 PCs were chosen for the dimensionality reduction, then the cell clusters were identified using the “FindNeighbors” and “FindClusters” functions.

In addition, Seurat V3 (V3.2.2) was also used for the integration analysis between CCs and embryonic SANPCs, the scRNA-seq data of which was obtained from SAN and adjacent atrial tissues of E13.5 embryos (GSE130461) (Li et al., 2019). In brief, embryonic cells which expressed genes less than 1000 and more than 6,000 were filtered firstly, PCA was then performed based on 2,000 variable genes. 20 PCs were chosen for the identity of cell clusters. Then cardiomyocyte (CM) cell cluster was identified using the cell markers reported in its study (Li et al., 2019). Accordingly, these CM cells were extracted for the sub-clustering analysis. The reported cell markers were used for identifying the SAN cells (SAN head and junction), atrium cells and C3 cell cluster. Finally, the SAN cells were extracted again and used for the integrated analysis with CCs data with the similar parameters described above (Fig. S6).

### Histology and immunohistochemistry

After anesthesia, the hearts of adult mice (8–12-week-old mice) were quickly removed, preserving the atrium and peripheral connections. After rinsing with cold PBS, the hearts were fixed with 4% paraformaldehyde (PFA, Sigma, USA) overnight at 4 °C. Then, the hearts were dehydrated in gradually increased concentrations of ethanol and then embedded in paraffin. Hearts were sectioned horizontally in 6 μm sections. The SAN of adult mice located in the posterior wall of the right atrium, in the intercaval region adjacent to the atrial muscle of the crista terminalis, extending from the superior to near the inferior vena cava. The SAN is surrounded by connective tissue, and the SAN artery passing through the area.

After deparaffinization, re-hydration and microwaving for antigen retrieval in citrate solution, paraffin-embedded heart slices were blocked with 10% goat serum (Invitrogen, USA) and incubated with primary antibody overnight at 4 °C. The following day, the slices were washed three times in PBST and then incubated with respective fluorescent secondary antibody (Invitrogen, USA) for 1h at room temperature. The slices were washed again in PBST and then stained with DAPI (Sigma, USA) to label the nuclei. Pictures were taken from a confocal microscope (Leica TCS SP8, USA).

### SANPCs immunofluorescence

Isolated SANPCs were attached to glass coverslips precoated with laminin. For immunofluorescence, the cells were fixed in 4% paraformaldehyde for 15 min at room temperature. After fixation, the SANPCs were permeabilized with 0.5% Triton X-100 for 10 min and then blocked with 4% normal goat serum in PBS for 1 h at room temperature. Next, the cells were incubated with primary antibodies overnight at 4 °C and washed with PBS (3 times for 5 min each). Then, the cells were incubated with Alexa Fluor conjugated second antibodies for 1h at room temperature. The nuclei were stained with 1 μg/mL DAPI for 5 min. The negative control of immunostaining in SANPCs was performed with secondary antibody incubation only (Fig. S9). The cells were imaged by confocal microscopy (Leica TCS SP8, USA) and analyzed using LAS X software.

### Field potential recordings from SAN tissues

SAN tissues from male C57BL/6 mice (8–12 weeks of age) were dissected from the region bordered by the crista terminalis and the superior and inferior vena cava. The SAN tissues were placed in a warm tissue bath with oxygenated Tyrode’s solution: 140 mmol/L NaCl, 5.4 mmol/L KCl, 1.2 mmol/L KH_2_PO_4_, 1.8 mmol/L CaCl_2_, 1.0 mmol/L MgCl_2_, 5.5 mmol/L glucose and 5 mmol/L HEPES (pH was adjusted to 7.4 with NaOH; bath temperature was warmed to 37 ± 0.5 °C). The field potential recordings were acquired as previously described (Zhu et al., 2009; Rolston et al., 2010; Bredeloux et al., 2020). Two shielded Ag/AgCl electrodes were positioned on the proximal part of superior and inferior vena cava to record the field potential. The tissues were equilibrated in the tissue bath until electrically stable (usually 40 min) and after that the spontaneous pacing frequency of the SAN tissues can maintain its pacing rate steadily for at least two hours. The electrical signals were amplified, digitized, and visualized during the experiment using LabChart7 (ADInstruments, Dunedin, New Zealand).

### Measurement of SANPCs’ Ca^2+^ transients

The isolated SANPCs were incubated in Tyrode’s solution were loaded with 0.5 μmol/L fluo-4 AM (AAT bioquest, USA) for 5–10 min at room temperature. After centrifuged, the supernatant was removed, and the pellet was re-suspended in the Tyrode’s solution. Then, the cells were placed in a heated chamber, mounted on a Leica DMI3000B inverted microscope, and visualized at 100× magnification. Fluorescent images were collected alternately at excitation wavelengths of 488nm with an emission wavelength range from 500 nm to 550 nm. The Ca^2+^ release of SANPCs was measured by IonOptix Imaging System (IonOptix Corporation, USA) and analyzed using the IonWizard™ v6.1 acquisition software.

### Antibodies

Antibodies included those against GLS (1:25 for immunohistochemistry, 1:100 for immunocytochemistry, ab93434, Abcam), GRINA (1:50 for immunohistochemistry, 1:100 for immunocytochemistry, ab216953, Abcam), GLUR3 (1:50 for immunohistochemistry, 1:100 for immunocytochemistry, ab232887, Abcam), mGluR1 (1:50 for immunohistochemistry, 1:100 for immunocytochemistry, NB110-39033SS, Novus), mGluR5 (1:50 for immunohistochemistry, 1:100 for immunocytochemistry, ab76316, Abcam), VGLUT1 (1:50 for immunohistochemistry, 48-2400, Invitrogen; 1:100 for immunocytochemistry, 135 303, Synaptic Systems), UNC13B (1:50 for immunohistochemistry, 1:100 for immunocytochemistry, TA308990, Origene), SNAP25 (1:50 for immunohistochemistry, GTX113839, Gene Tex; 1:100 for immunocytochemistry, ab31281, Abcam), GAD1 (1:50 for immunohistochemistry, 1:100 for immunocytochemistry, ab26116, Abcam), SYT11 (1:50 for immunohistochemistry, 1:100 for immunocytochemistry, PA5-96970, Invitrogen), CAST (1:50 for immunohistochemistry, 1:100 for immunocytochemistry, PA5-87352, Invitrogen), and HCN4 (1:50 for immunohistochemistry, 1:100 for immunocytochemistry, SAB5200035, Sigma; SMC-320, StressMarq Biosciences). Alexa Fluor conjugated secondary antibodies were purchased from Abcam.

### Statistical analysis

Statistical analyses were performed with GraphPad Prism 8.0 software. The data are presented as mean ± SEM. No statistical methods were used to predetermine sample size. Normality was tested with the Kolmogorov-Smirnov test. Groups were compared using an unpaired Student’s *t*-test (two sided) and mixed-effects model in each of the specific experimental designs presented in the figures. *P* < 0.05 was considered as statistically significant.

## Supplementary information

Below is the link to the electronic supplementary material.

Supplementary material 1 (PDF 1166 kb)
