# Supplementary material for: Sinoatrial node pacemaker cells share dominant biological properties with glutamatergic neurons
Source: Protein Cell. 2021 Feb 6;12(7):545–56. doi: 10.1007/s13238-020-00820-9 (PMC8225718; doi:10.1007/s13238-020-00820-9)
Supplement: Supplementary file 1 — Supplementary material 1 (PDF 1166 kb) [file 13238_2020_820_MOESM1_ESM.pdf]

SUPPLEMENTAL MATERIAL

Sinoatrial node pacemaker cells share dominant biological properties with glutamatergic neurons

Table of contents:

Figure S1-S9

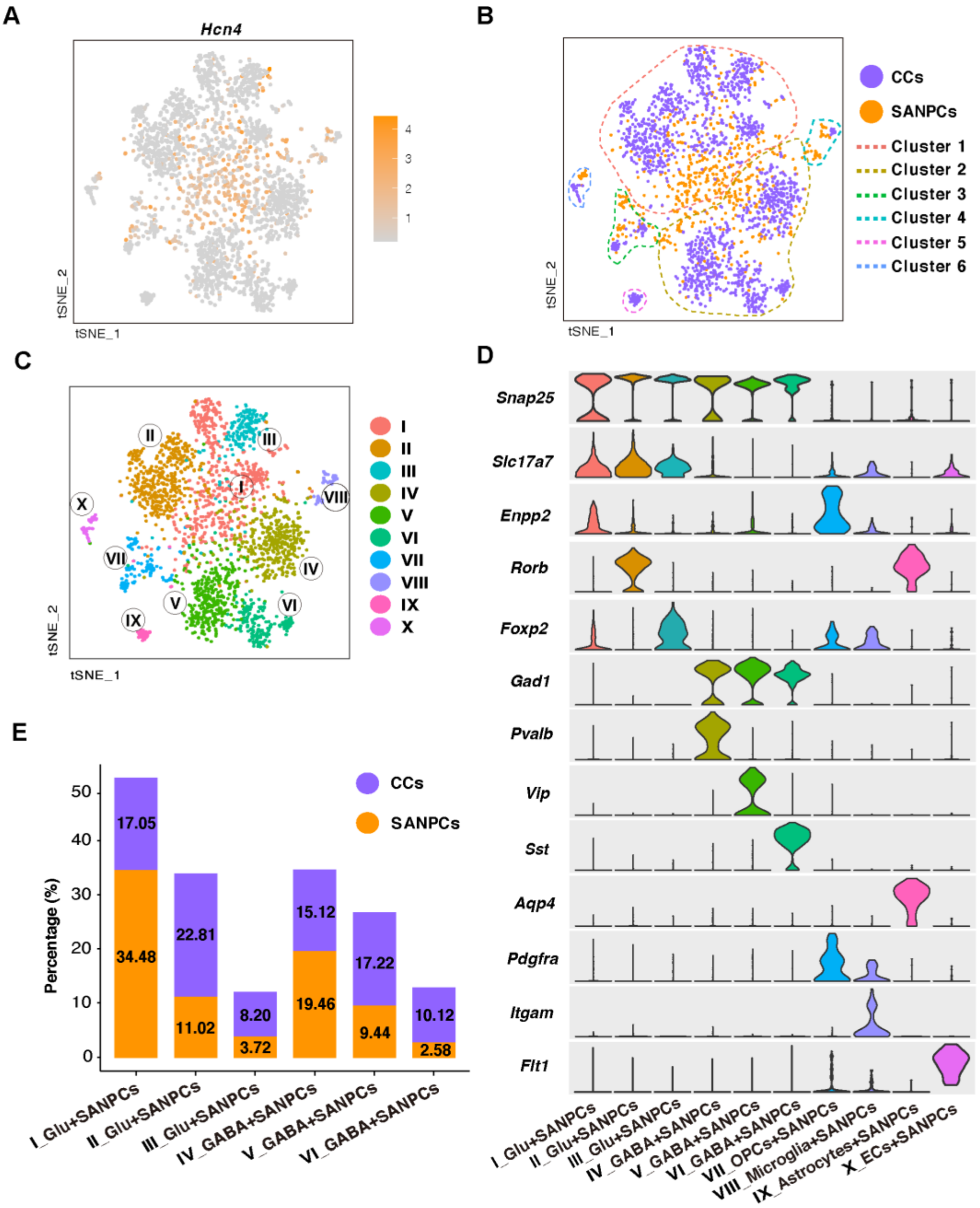

Figure S1. Co-clustering of SANPCs with CCs at single-cell transcriptomic level. (A) Expression of *Hcn4* confirmed the cell types of SANPCs. (B) Distribution of SANPCs and CCs in each cluster. Each cell type was combined with SANPCs and CCs. (C) Clustering analysis showed subpopulations in Cluster 1 (Subcluster I-III) and Cluster 2 (Subcluster IV-VI). (D) Violin plot showed expression of canonical cell markers in each subclusters: *Snap25* (pan-neuronal), *Slc17a7* (pan-glutamatergic); *Enpp2*, *Rorb and Foxp2* (glutamatergic); *Gad1* (pan-GABAergic); *Vip*, *Sst* and *Pvalb* (GABAergic); *Aqp4* (astrocytes); *Pdgfra* (OPCs); *Itgam* (microglia) and *Flt1* (ECs). (E) Distribution percentages of SANPCs and CCs in the subpopulations of Cluster 1 and Cluster 2. Glu, glutamatergic neuron; GABA, GABAergic neuron.

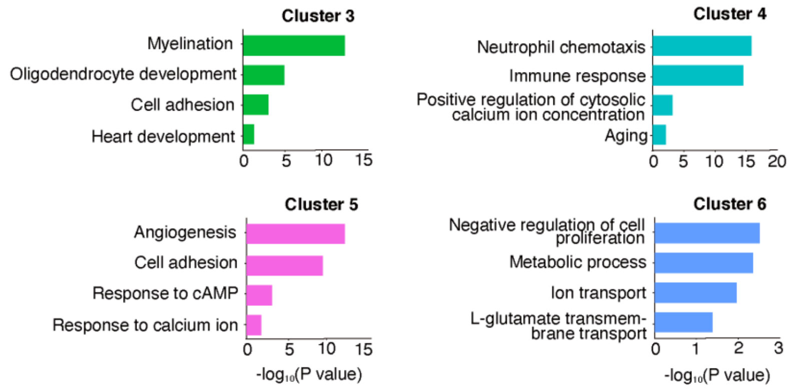

Figure S2. GO terms of non-neuronal clusters (Cluster 3-6).

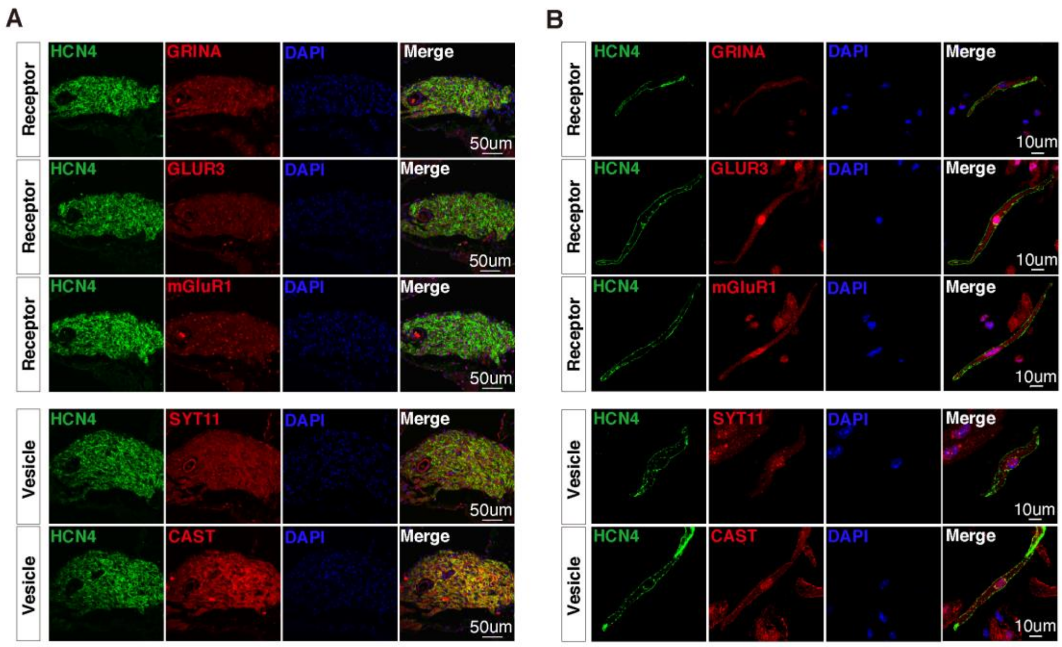

Figure S3. Immunofluorescence staining detected the expression of the glutamatergic neurotransmitter receptors and the proteins related to synaptic vesicle transporters in SAN tissue (A) **and single SANPC** (B).

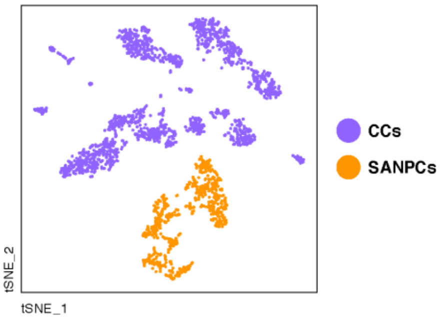

Figure S4. t-SNE plot of uncorrected datasets of SANPCs and CCs.

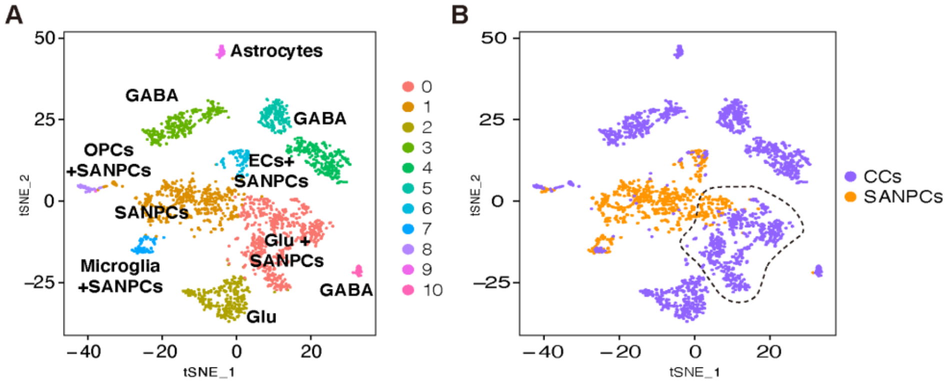

Figure S5. Integrated clustering analysis of SANPCs and CCs using Seurat V3 software. (A) t-SNE plot showed different cell types. SANPCs and CCs were co-clustered within Cluster 0, and SANPCs showed a close relationship with glutamatergic neuron clusters. (B) The distribution of SANPCs and CCs. Cells in Cluster 0 were marked with black circle.

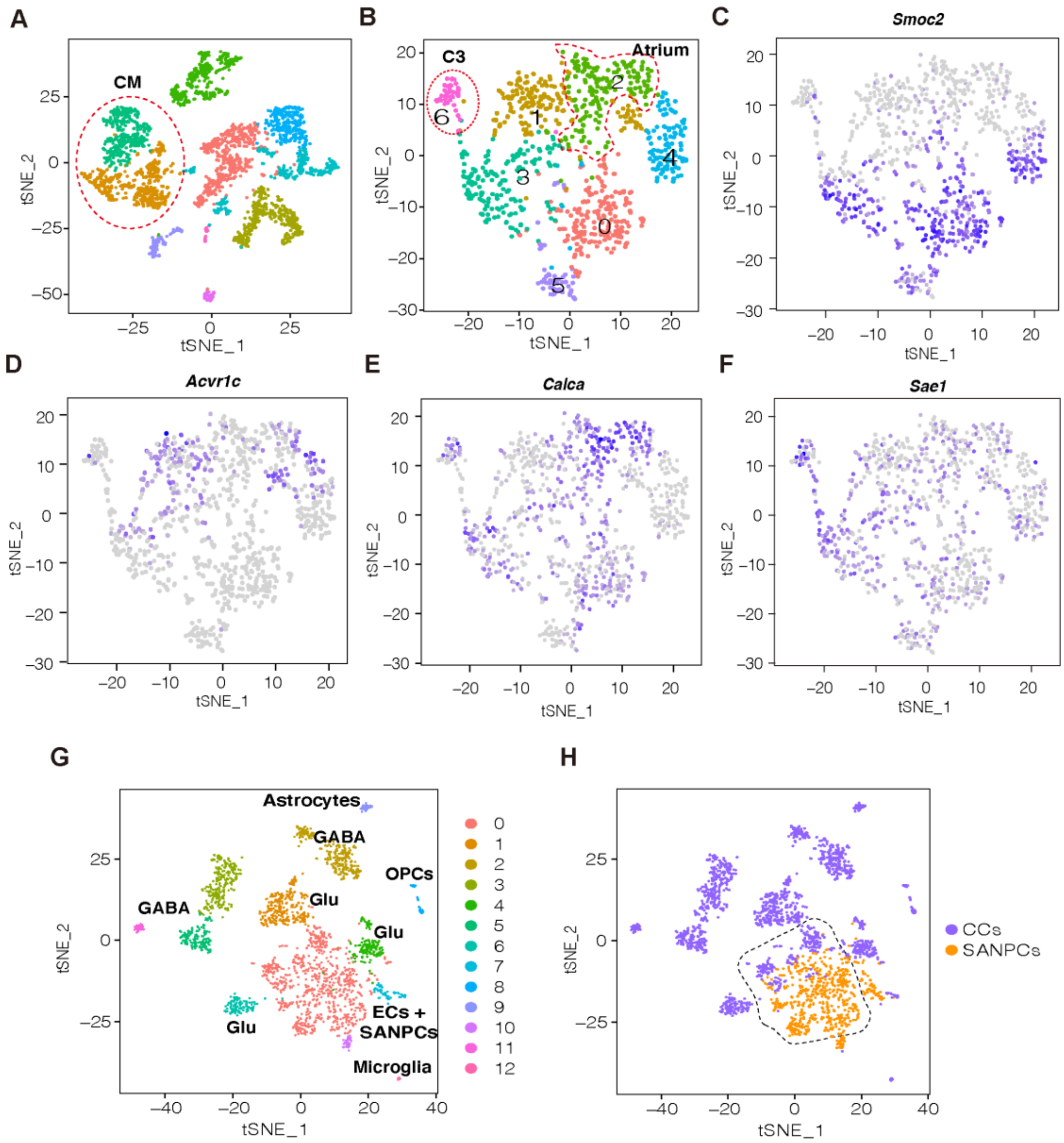

Figure S6. **Integrated clustering analysis of embryonic SANPCs with CCs using Seurat V3 software.** (A) t-SNE plot showed the different cell types of mouse E13.5 SAN and atrial cells. Cardiomyocyte (CM) clusters were confirmed using the expression of marker genes including *Hcn4*, *Shox2* and *Tbx3* as reported. CM cluster was marked with red circle. (B) Sub-clustering analysis of CM cells. Cells in CM cluster were extracted for the sub-clustering. Non-SAN cells (Atrium and C3 cells, marked with red circle) were identified using the expression of reported marker genes. (C-F) Feature plots showed the expression of marker genes of CM subclusters, including SAN head (*Smoc2*, Cluster 0, 3, 4, 5), SAN junction (*Acvr1c*, Cluster 1), Atrium (*Calca*, Cluster 2) and C3 (*Sae1*, Cluster 6). Cells of SAN head and SAN junction (Cluster 0, 1, 3, 4, 5) were identified as embryonic SANPCs. (G) t-SNE plot showed diverse cell types of CCs and embryonic SANPCs. Identified SANPCs from CM cells were extracted and used for clustering analysis with CCs. Most SANPCs were co-clustered with a part of glutamatergic neurons (Cluster 0). (H) The distribution of SANPCs and CCs. Cells in Cluster 0 (G) were marked with black circle.

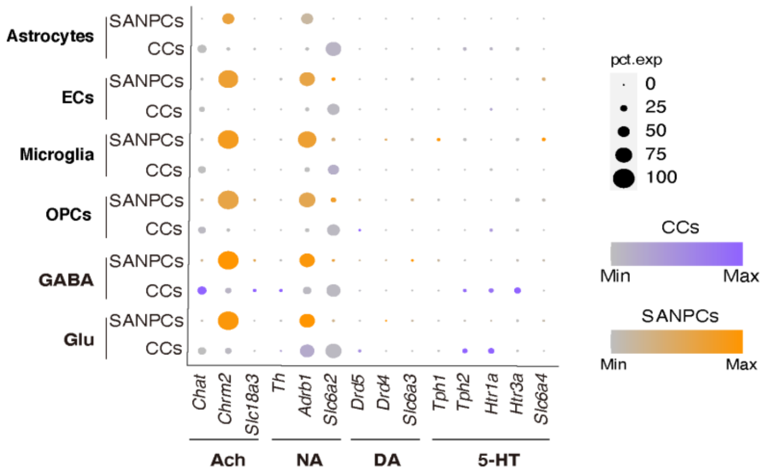

Figure S7. Expression of functional genes related to different neurotransmitter systems in SANPCs and CCs in each cluster. The size of the dots showed the percentage of cells which expressed the genes in clusters. The brightness of colors showed the relative expression level of genes. Glu, glutamatergic neuron; GABA, GABAergic neuron; OPCs, oligodendrocyte precursor cells; ECs, endothelial cells; Ach, acetylcholine; NA, noradrenaline; DA, dopamine; 5-HT, serotonin.

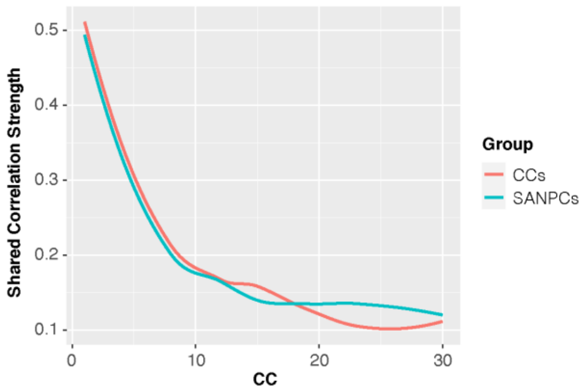

Figure S8. MetageneBicor plot of SANPCs and CCs datasets. 15 CC components were chosen for the subsequent analysis.

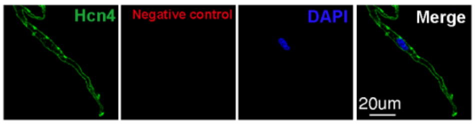

Figure S9. The negative control of immunofluorescence staining in Fig. 2B. Immunostaining was performed with secondary antibody incubation only.
